# Bazex Syndrome as a Cutaneous Marker of Occult Malignancy: A Report of Two Cases

**DOI:** 10.7759/cureus.116363

**Published:** 2026-09-16

**Authors:** Beatriz Dias Silva, Inês Fernandes, Sofia Camões, Filipa Reis, João Olivério Ribeiro

**Affiliations:** 1 Department of Internal Medicine, Unidade Local de Saúde de Viseu Dão-Lafões, Viseu, PRT

**Keywords:** acrokeratosis paraneoplastica, bazex syndrome, neuroendocrine carcinoma, occult malignancy, oesophageal squamous cell carcinoma, paraneoplastic dermatosis

## Abstract

Acrokeratosis paraneoplastica (Bazex syndrome) is a rare paraneoplastic dermatosis characterized by psoriasiform lesions predominantly affecting acral sites and is most commonly associated with squamous cell carcinomas of the upper aerodigestive tract. Cutaneous manifestations may precede the diagnosis of the underlying malignancy, making their recognition an important diagnostic clue. We report two cases of Bazex syndrome in older men presenting with characteristic acral and facial psoriasiform lesions. In both patients, skin biopsy revealed nonspecific histopathological findings, while the clinical pattern prompted investigation for an underlying malignancy. The first patient was subsequently diagnosed with metastatic oesophageal squamous cell carcinoma. In the second patient, investigation revealed two synchronous malignancies: a large-cell neuroendocrine carcinoma and an oesophageal squamous cell carcinoma. These cases emphasize the diagnostic value of recognizing the clinical pattern of Bazex syndrome as a marker of underlying malignancy. In patients presenting with compatible acral psoriasiform lesions, particularly older individuals with relevant cancer risk factors, this distinctive clinical pattern should prompt systematic investigation for an occult malignancy.

## Introduction

Paraneoplastic dermatoses represent a heterogeneous group of cutaneous manifestations associated with internal malignancies and may provide an important clinical clue to an otherwise occult neoplasm. Their recognition is particularly relevant when cutaneous findings precede other signs or symptoms of malignancy, sometimes by several months, thereby prompting diagnostic investigation before the underlying malignancy becomes otherwise clinically apparent [1-4]. Importantly, this does not necessarily imply diagnosis at an early oncological stage, as the malignancy may already be advanced when the cutaneous manifestations are recognized.

Acrokeratosis paraneoplastica, or Bazex syndrome (BS), is a rare paraneoplastic dermatosis characterized by erythematous to violaceous, scaly, or psoriasiform lesions with a distinctive acral distribution, typically involving the ears, nose, hands, and feet [1-4]. The syndrome predominantly affects older men, typically presenting in the sixth and seventh decades of life, and is most strongly associated with squamous cell carcinomas of the upper aerodigestive tract, particularly those involving the pharynx, larynx, and oesophagus [1-3]. Nevertheless, its association is not restricted to these malignancies, and BS has been reported in patients with a broader spectrum of solid and haematological neoplasms, including pulmonary, genitourinary, and neuroendocrine tumours [1,2,4-6]. Although its pathogenesis remains incompletely understood, proposed mechanisms include tumour-derived growth factors, immune-mediated responses and alterations in epidermal growth factor receptor signalling [1,4].

Despite its characteristic dermatological presentation, the diagnosis of BS can be challenging because the eruption may resemble more common inflammatory dermatoses, particularly psoriasis and eczema [1,2,4,7]. The diagnosis is primarily clinical, based on recognition of its characteristic morphology and distribution in the appropriate clinical context [1,2,4]. Histopathological findings are generally nonspecific, and skin biopsy is therefore mainly useful for excluding alternative diagnoses rather than confirming BS [2,5,8]. We report two cases of BS in which characteristic cutaneous manifestations provided an important clue to underlying cancer, including one patient in whom two histologically distinct malignancies were identified synchronously.

## Case presentation

Case 1

A 65-year-old man with a 45-pack-year smoking history, no history of significant alcohol consumption, and no other relevant medical history was referred to the Internal Medicine outpatient clinic for a four-month history of bilateral, symmetrical, pruritic, and scaly palmar lesions, with additional involvement of the nose and ears (Figure 1). He denied dysphagia, odynophagia, weight loss, or other constitutional symptoms. No lesions were observed elsewhere on the body, and the patient denied previous dermatological treatments. A skin biopsy was performed, revealing mild hyperkeratosis with focal compact parakeratosis. Although nonspecific, these histopathological findings were considered compatible with acrokeratosis paraneoplastica in the appropriate clinical context. Importantly, the characteristic acral and facial distribution of the lesions, together with the patient’s age and significant smoking history, raised suspicion of BS as a paraneoplastic manifestation. The patient was therefore admitted for further diagnostic investigation.

**Figure 1:**
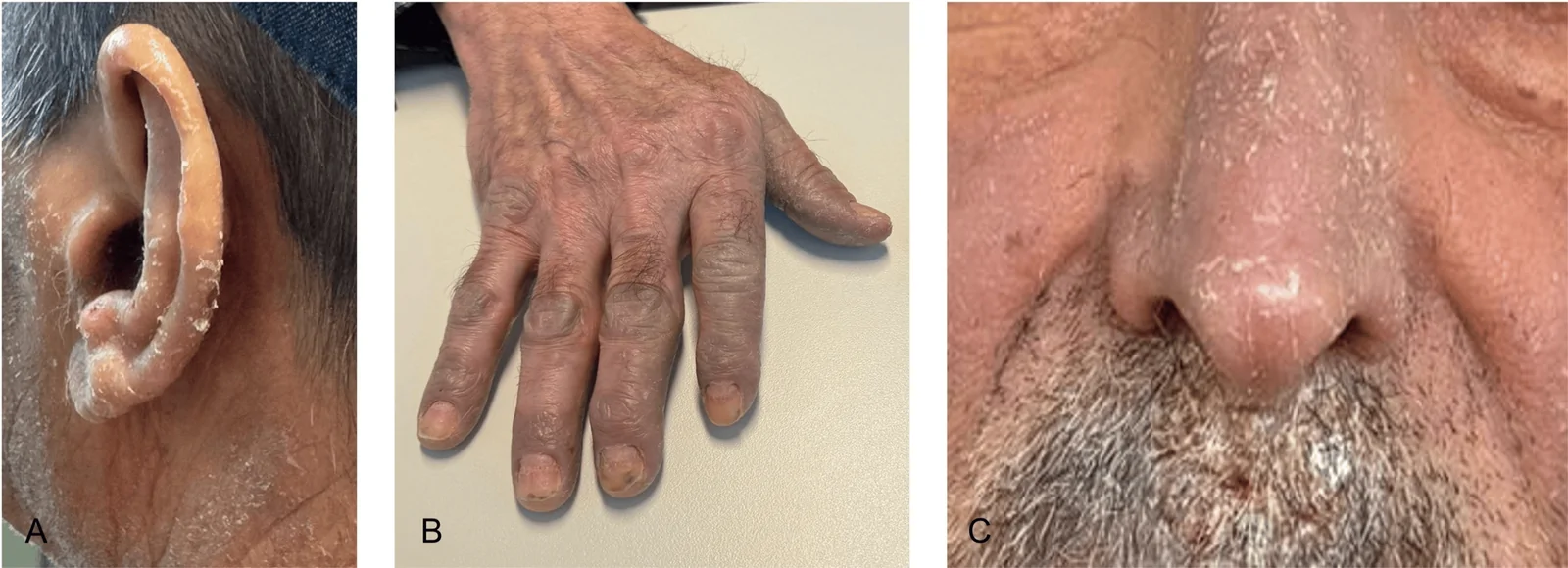
Cutaneous manifestations of Bazex syndrome in Case 1 (A–C) Representative clinical photographs showing the characteristic acral distribution of the cutaneous lesions: (A) scaly lesions involving the auricle; (B) scaly lesions involving the palmar surface of the hand and fingers; (C) scaly lesions involving the nasal and perinasal regions.

During hospitalization, upper gastrointestinal endoscopy revealed two vegetative oesophageal lesions. Histopathological examination of the endoscopic biopsies confirmed a poorly differentiated squamous cell carcinoma, most likely originating in the distal oesophagus with involvement of the middle oesophagus. As part of the staging workup, contrast-enhanced computed tomography of the chest, abdomen, and pelvis revealed multiple blastic bone lesions highly suggestive of metastatic dissemination.

Despite diagnostic evaluation prompted by the suspicion of an occult malignancy, metastatic bone disease was already present at the time of the cancer diagnosis. The patient was subsequently evaluated by General Surgery and Medical Oncology and, given the advanced stage of the disease, a palliative treatment strategy was adopted. Topical and systemic corticosteroids were prescribed for symptomatic control of the pruritus, with only partial improvement, while the cutaneous lesions persisted.

Case 2

A 72-year-old man with no relevant medical history, no history of smoking, and a history of excessive alcohol consumption, with abstinence for approximately four years, was referred to the Internal Medicine outpatient clinic for investigation of epigastric lymphadenopathy incidentally identified on abdominal ultrasonography. He denied dysphagia, odynophagia, weight loss, or any other constitutional symptoms. Clinical examination revealed multiple bilateral, symmetrical, erythematous, and scaly lesions involving the palms, with additional involvement of the nose and ears, which had developed approximately two months earlier and were not associated with pruritus (Figure 2). No lesions were observed elsewhere on the body, and the patient denied previous dermatological treatments. The characteristic distribution of the cutaneous lesions, together with the patient’s age, previous excessive alcohol consumption, and the presence of unexplained lymphadenopathy, raised suspicion of BS as a paraneoplastic manifestation and prompted hospital admission for further investigation.

**Figure 2:**
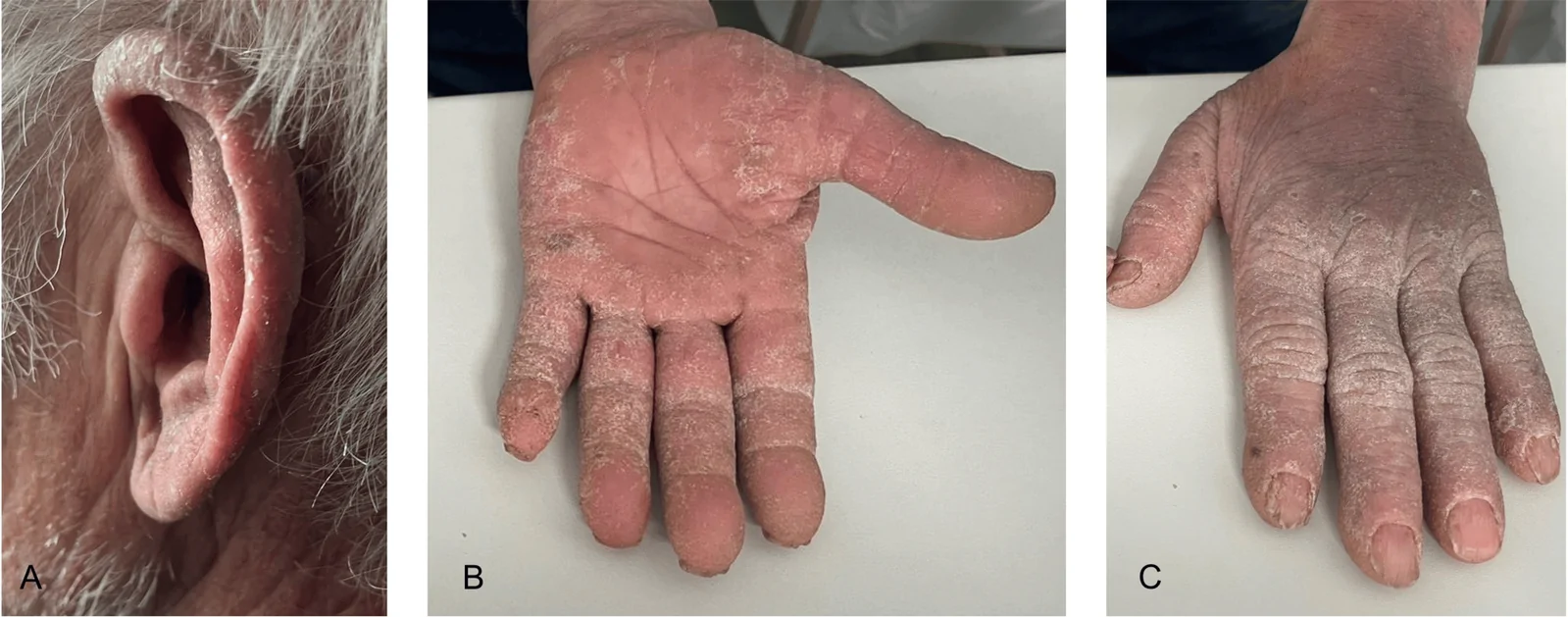
Cutaneous manifestations of Bazex syndrome in Case 2 (A–C) Representative clinical photographs showing the characteristic acral distribution of the cutaneous lesions: (A) scaly lesions involving the auricle; (B) erythematous and scaly lesions involving the palmar surfaces of the hands and fingers; (C) erythematous and scaly lesions involving the dorsal surfaces of the hands and fingers.

Skin biopsy revealed histopathological findings similar to those observed in Case 1, with mild hyperkeratosis and focal compact parakeratosis. Biopsy of the enlarged lymph nodes subsequently revealed a large-cell neuroendocrine carcinoma. During the same diagnostic workup, upper gastrointestinal endoscopy identified an oesophageal lesion located 27 cm from the incisors. Histopathological examination confirmed a squamous cell carcinoma of the oesophagus. Thus, the diagnostic workup identified two synchronous, histologically distinct malignancies.

The patient was subsequently referred to Medical Oncology and General Surgery for therapeutic evaluation. During the interval between diagnosis and oncological assessment for treatment initiation, his general condition deteriorated significantly. Following multidisciplinary assessment, he was deemed unsuitable for cancer-directed treatment and was therefore referred for palliative care. Further information regarding the evolution of the cutaneous lesions during palliative management was not available.

## Discussion

BS, or acrokeratosis paraneoplastica, is a rare paraneoplastic dermatosis classically associated with squamous cell carcinomas of the upper aerodigestive tract, particularly those arising from the pharynx, larynx, and oesophagus [1,2,9]. Nevertheless, subsequent reports have substantially broadened the spectrum of associated malignancies, including pulmonary, genitourinary, haematological, and neuroendocrine neoplasms [1-6]. The two cases presented here reflect this oncological spectrum: the first represents the classical association with oesophageal squamous cell carcinoma, whereas the second is distinguished by the synchronous diagnosis of an oesophageal squamous cell carcinoma and a large-cell neuroendocrine carcinoma.

The pathogenesis of BS remains incompletely understood, and several mechanisms have been proposed. These include immune-mediated cross-reactivity between tumour and cutaneous antigens, deposition of immunoglobulins at the basement membrane and the release of cytokines or growth factors by tumour cells, particularly epidermal growth factor and transforming growth factor-alpha [2-4]. None of these mechanisms has been definitively established, and the pathogenesis is likely multifactorial. The frequent correlation between tumour activity and the course of cutaneous manifestations, with improvement of skin lesions following successful oncological treatment in many patients, further supports the paraneoplastic nature of the syndrome [1,2,4].

Clinically, BS predominantly affects men older than 50 years and is characterized by erythematous to violaceous, scaly, or hyperkeratotic lesions with a distinctive acral distribution [1-3]. The ears, nose, fingers, and toes are typically affected early in the disease, with subsequent extension to the palms and soles and, in more advanced stages, to the elbows, knees, and trunk [1-4]. Pruritus and nail abnormalities may also occur, although pruritus is not invariably present [2,4], as illustrated by contrasting presentations of our patients: the first experienced pruritic lesions requiring symptomatic treatment, whereas the second had similar acral eruption without pruritus. Importantly, the cutaneous eruption frequently precedes the diagnosis of the underlying malignancy, making recognition of this characteristic syndrome particularly relevant in patients without a known cancer diagnosis [1-3,9]. Both patients displayed this typical clinical pattern, with bilateral and symmetrical involvement of the hands, ears, and nose before the associated malignancies were identified. Notably, neither patient exhibited the full spectrum of classically described cutaneous involvement.

The diagnosis of BS is primarily clinical and relies on recognition of its characteristic morphology and distribution in the appropriate oncological context. Histopathological findings are generally nonspecific and may include hyperkeratosis, parakeratosis, acanthosis, spongiosis and a variable inflammatory infiltrate [2,4,5,8]. Accordingly, skin biopsy does not establish the diagnosis of BS but may be valuable in excluding alternative conditions with a similar clinical appearance. The differential diagnosis includes psoriasis, chronic eczema and superficial fungal infections, among other erythematous and scaly dermatoses [2,5,8]. In both patients, the characteristic acral and facial distribution of the lesions, together with relevant cancer risk factors, raised clinical suspicion of BS and prompted investigation for an underlying malignancy, which was subsequently identified.

The oncological findings in our patients further illustrate both the classical and less common associations of the syndrome. In the first case, the cutaneous manifestations preceded the diagnosis of a poorly differentiated oesophageal squamous cell carcinoma, consistent with one of the malignancies most frequently associated with BS [1,2,10]. Despite investigation prompted by the dermatological presentation, metastatic bone disease was already present at the time of diagnosis. This underscores an important limitation of BS as an early cancer marker: although the cutaneous manifestations may precede cancer detection, their recognition does not necessarily correspond to an early stage of the underlying malignancy.

The second case illustrates a more unusual scenario, as two synchronous, histologically distinct malignancies were identified during the diagnostic workup. While the oesophageal squamous cell carcinoma represents a well-established association with BS [10], neuroendocrine neoplasms have also been linked to the syndrome [6]. Consequently, although the oesophageal malignancy represents the more typical association in our patient, the concomitant large-cell neuroendocrine carcinoma cannot be excluded as a potential driver of the cutaneous manifestations. The coexistence of both neoplasms therefore precludes reliable attribution of BS to either tumour individually. Moreover, because effective oncological treatment could not be undertaken, the subsequent behaviour of the skin lesions could not be used to clarify this association.

Given the strong association between BS and internal malignancy, clinical suspicion of this syndrome should prompt investigation for an underlying neoplasm, even in the absence of cancer-related symptoms. Evaluation should be individualized according to the patient’s age, clinical findings and risk factors, with particular attention to the upper aerodigestive tract, and may include cross-sectional imaging and endoscopic evaluation [1,2,4,7].

Management of BS is primarily directed towards treatment of the underlying malignancy. Improvement or complete regression of the cutaneous lesions following successful tumour treatment is frequently reported, whereas persistence or recurrence of the eruption may accompany persistent or recurrent malignancy [1,2,4,11,12]. Skin-directed therapies, including topical or systemic corticosteroids, keratolytic agents, vitamin D analogues, retinoids, and phototherapy, have been used with variable efficacy and are primarily intended for symptomatic control [2,4,11]. In our first patient, topical and systemic corticosteroids were used for symptomatic control of pruritus, resulting in only partial improvement. Neither patient underwent effective cancer-directed treatment, preventing assessment of whether tumour control would have resulted in regression of the cutaneous manifestations.

## Conclusions

BS is an uncommon but clinically significant paraneoplastic dermatosis whose cutaneous manifestations may precede the diagnosis of an underlying malignancy. These cases highlight the diagnostic value of recognizing its characteristic morphology and acral distribution, which may provide an important clinical clue to an otherwise occult neoplasm. Awareness of this distinctive clinical pattern should therefore prompt a thorough search for an underlying malignancy, facilitating its recognition and appropriate oncological assessment.
